# Hemorrhagic and ischemic stroke in patients with coronavirus disease 2019: incidence, risk factors, and pathogenesis - a systematic review and meta-analysis

**DOI:** 10.12688/f1000research.42308.1

**Published:** 2021-01-19

**Authors:** Syahrul Syahrul, Helnida Anggun Maliga, Muhammad Ilmawan, Marhami Fahriani, Sukamto S. Mamada, Jonny Karunia Fajar, Andri Frediansyah, Faza Nabila Syahrul, Imran Imran, Salim Haris, Aldy Safruddin Rambe, Talha Bin Emran, Ali A. Rabaan, Ruchi Tiwari, Kuldeep Dhama, Firzan Nainu, Endang Mutiawati, Harapan Harapan

**Affiliations:** 1 Department of Neurology, School of Medicine, Universitas Syiah Kuala, Banda Aceh, Aceh, 23111, Indonesia; 2 Department of Neurology, Dr. Zainoel Abidin Hospital, Banda Aceh, Aceh, 23111, Indonesia; 3 Faculty of Medicine, Universitas Brawijaya, Malang, East Java, 65117, Indonesia; 4 Medical Research Unit, School of Medicine, Universitas Syiah Kuala, Banda Aceh, Aceh, 23111, Indonesia; 5 Faculty of Pharmacy, Hasanuddin University, Makassar, South Sulawesi, 90245, Indonesia; 6 Brawijaya Internal Medicine Research Center, Department of Internal Medicine, Faculty of Medicine, Universitas Brawijaya, Malang, East Java, 65145, Indonesia; 7 Research Division for Natural Product Technology (BPTBA), Indonesian Institute of Sciences (LIPI), Wonosari, 55861, Indonesia; 8 Department of Neurology, Faculty of Medicine, Universitas Indonesia, Jakarta, 10430, Indonesia; 9 Department of Neurology, Faculty of Medicine, Universitas Sumatera Utara, Medan, North Sumatra, 20155, Indonesia; 10 Department of Pharmacy, BGC Trust University Bangladesh, Chittagong-4381, Bangladesh; 11 Molecular Diagnostic Laboratory, Johns Hopkins Aramco Healthcare, Dhahran, 31311, Saudi Arabia; 12 Department of Veterinary Microbiology and Immunology, College of Veterinary Sciences, UP Pandit Deen Dayal Upadhayay Pashu Chikitsa Vigyan Vishwavidyalay Evum Go-Anusandhan Sansthan (DUVASU), Mathura, Uttar Pradesh, 281 001, India; 13 Division of Pathology, ICAR-Indian Veterinary Research Institute, Izatnagar, Uttar Pradesh, 243122, India; 14 Department of Microbiology, School of Medicine, Universitas Syiah Kuala, Banda Aceh, Aceh, 23111, Indonesia; 15 Tropical Disease Centre, School of Medicine, Universitas Syiah Kuala, Banda Aceh, Aceh, 23111, Indonesia

**Keywords:** COVID-19, haemorrhagic stroke, ischemic stroke, meta-analysis, pathogenesis, SARS-CoV-2, systematic review

## Abstract

**Background**: In this study, we aimed to determine the global prevalence, chronological order of symptom appearance, and mortality rates with regard to hemorrhagic and ischemic stroke in patients with coronavirus disease 2019 (COVID-19) and to discuss possible pathogeneses of hemorrhagic and ischemic stroke in individuals with the disease.

**Methods**: We searched the PubMed, Scopus, and Web of Science databases for relevant articles published up to November 8, 2020. Data regarding study characteristics, hemorrhagic stroke, ischemic stroke, and COVID-19 were retrieved in accordance with the PRISMA guidelines. The Newcastle-Ottawa scale was used to assess the quality of the eligible studies. The pooled prevalence and mortality rate of hemorrhagic and ischemic stroke were calculated.

**Results**: The pooled estimate of prevalence of hemorrhagic stroke was 0.46% (95% CI 0.40%–0.53%; *I ^2^*=89.81%) among 67,155 COVID-19 patients and that of ischemic stroke was 1.11% (95% CI 1.03%–1.22%; *I ^2^*=94.07%) among 58,104 COVID-19 patients. Ischemic stroke was more predominant (incidence: 71.58%) than hemorrhagic stroke (incidence: 28.42%) in COVID-19 patients who experienced a stroke. In COVID-19 patients who experienced a stroke, hospital admission with respiratory symptoms was more commonly reported than that with neurological symptoms (20.83% for hemorrhagic stroke and 5.51% for ischemic stroke versus **6.94% for hemorrhagic stroke and 5.33% for ischemic stroke, respectively). The pooled mortality rate of COVID-19 patients who experienced a hemorrhagic and ischemic stroke was 44.72% (95% CI 36.73%–52.98%) and 36.23% (95% CI 30.63%–42.24%), respectively.

**Conclusions**: Although the occurrence of hemorrhagic and ischemic stroke is low, the mortality rates of both stroke types in patients with COVID-19 are concerning, and therefore, despite several potential pathogeneses that have been proposed, studies aimed at definitively elucidating the mechanisms of hemorrhagic and ischemic stroke in individuals with COVID-19 are warranted.

**PROSPERO registration: **CRD42020224470 (04/12/20)

## Introduction

Coronavirus disease 2019 (COVID-19), which is caused by severe acute respiratory syndrome coronavirus 2 (SARS-CoV-2) infection, has become a global human pandemic that is believed to have begun in late December 2019. The disease quickly spread to 217 countries, infected more than 82 million individuals, and has caused more than 1.8 million deaths as of December 30, 2020 ^1^. Moreover, the second wave of this pandemic remains ongoing in various countries ^2^. Numerous treatment strategies and drugs have been proposed ^3– 5^; however, a definitive therapy or treatment for COVID-19 has not yet been announced by the World Health Organization ^6^. SARS-CoV-2 is a novel coronavirus, which is reported to have originated initially from an animal source ^7^. The mortality rate of SARS-CoV-2 infection is the lowest among the infections caused by other members of the coronavirus family that have previously infected humans, including severe acute respiratory syndrome coronavirus (SARS-CoV) and Middle East respiratory syndrome coronavirus (MERS-CoV) ^8, 9^. However, SARS-CoV-2 has a higher reproduction rate (R _0_) and thus a higher transmission rate than SARS-CoV and MERS-CoV ^10^.

The majority of individuals infected with SARS-CoV-2 are generally asymptomatic, although the most common symptoms of COVID-19 include dry cough, fever, dyspnea, chest pain, headache, and muscle ache ^11, 12^. The issue of hypercoagulability-related thrombotic vascular events in those infected with SARS-CoV-2 is emerging ^13, 14^. Evidence suggests that COVID-19 patients may experience increased rates of thromboembolism, as high as 15%–26% ^15^. In addition, another concern is the increased risk of a hemorrhagic stroke among COVID-19 patients ^16– 18^. The World Stroke Organization has reported that COVID-19 increases the risk for an ischemic stroke by approximately 5% (95% confidence interval (CI) 2.8%–8.7%) ^19^. Other possible explanations for the occurrence of ischemic stroke in COVID-19 patients include reduced angiotensin (ANG) (1-7) synthesis ^20^, cardioembolism ^21, 22^, hyperviscosity ^23, 24^, and an induced hypercoagulative state ^25, 26^. Discussions surrounding the possible mechanism(s) underlying hemorrhagic stroke in COVID-19 patients have included the expression of angiotensin-converting enzyme 2 (ACE2), immunity, inflammation, endothelial dysfunction at the blood-brain-barrier (BBB), aging, stress, and anxiety ^27^.

The aims of our present study were to determine the global incidence of ischemic and hemorrhagic stroke in patients with COVID-19; determine the mortality rate of ischemic and hemorrhagic stroke in individuals with COVID-19; assess the frequency of symptoms that lead to hospital admission among COVID-19 patients who have experienced an ischemic or a hemorrhagic stroke; assess the risk factors for ischemic and hemorrhagic stroke in COVID-19; assess the association between ischemic and hemorrhagic stroke and the severity of COVID-19; and assess the association between ischemic and hemorrhagic stroke and mortality in COVID-19. In addition, we also sought to propose possible pathogeneses of ischemic and hemorrhagic stroke in individuals with SARS-CoV-2 infection.

## Methods

### Registration and protocol

This systematic review and meta-analysis identified the stroke proportion among COVID-19 confirmed cases. We followed the Preferred Reporting Items for Systematic Reviews and Meta-analyses (PRISMA) recommendation to search electronic databases (see completed checklist ^28^) ^29^. The protocol of this study was registered in PROSPERO ( CRD42020224470) on 4 ^th^ December 2020.

### Eligibility criteria of studies 

The inclusion criteria were articles written in English which identified stroke as a comorbidity among randomly sampled COVID-19 cases. All case reports, case series, editorials, reviews, commentaries, and studies in targeted specific groups, such as in children were excluded.

### Information sources and search strategy

The systematic searches were conducted in three databases ( PubMed, Scopus, and Web of Science) to identify the potential articles as of November 8 ^th^, 2020. The search criteria were as follows. Scopus (TITLE("SARS-CoV-2" OR "COVID-19" OR "Wuhan coronavirus" OR "Wuhan virus" OR "novel coronavirus" OR "nCoV" OR "severe acute respiratory syndrome coronavirus 2" OR "coronavirus disease 2019 virus" OR "2019-nCoV" OR "2019 novel coronavirus" OR "severe acute respiratory syndrome coronavirus 2" OR "coronavirus" OR "coronaviruses" OR "SARS 2" OR "2019-nCoV acute respiratory disease" OR "novel coronavirus pneumonia" OR "COVID") AND ALL("Stroke " OR "cerebrovascular disorders" OR "brain ischemia" OR "brain haemorrhage" OR "cerebrovascular accident" OR "intracerebral haemorrhage" OR "subarachnoid haemorrhage" OR "transient ischemic attack" OR "brain attack" OR "cerebral embolism" OR "cerebral thrombosis" OR "cerebral haemorrhage" OR "cerebrovascular insult" OR "intraparenchymal haemorrhage" OR "intraventricular haemorrhage" OR "cerebral hypoperfusion" OR "brain infarct" OR "cerebral infarct"). Web of Science (TITLE("SARS-CoV-2" OR "COVID-19" OR "Wuhan coronavirus" OR "Wuhan virus" OR "novel coronavirus" OR "nCoV" OR "severe acute respiratory syndrome coronavirus 2" OR "coronavirus disease 2019 virus" OR "2019-nCoV" OR "2019 novel coronavirus" OR "severe acute respiratory syndrome coronavirus 2" OR "coronavirus" OR "coronaviruses" OR "SARS 2" OR "2019-nCoV acute respiratory disease" OR "novel coronavirus pneumonia" OR "COVID") AND ALL=("stroke" OR "cerebrovascular Disorders" OR "brain ischemia" OR "brain haemorrhage" OR "cerebrovascular accident" OR "intracerebral haemorrhage" OR "subarachnoid haemorrhage" OR "transient ischemic attack" OR "brain attack" OR "cerebral embolism" OR "cerebral thrombosis" OR "cerebral haemorrhage" OR "cerebrovascular insult" OR "intraparenchymal haemorrhage" OR "intraventricular haemorrhage" OR "cerebral hypoperfusion" OR "brain infarct" OR "cerebral infarct"). PubMed (TITLE("SARS-CoV-2" OR "COVID-19" OR "Wuhan coronavirus" OR "Wuhan virus" OR "novel coronavirus" OR "nCoV" OR "severe acute respiratory syndrome coronavirus 2" OR "coronavirus disease 2019 virus" OR "2019-nCoV" OR "2019 novel coronavirus" OR "severe acute respiratory syndrome coronavirus 2" OR "coronavirus" OR "coronaviruses" OR "SARS 2" OR "2019-nCoV acute respiratory disease" OR "novel coronavirus pneumonia" OR "COVID") AND ("stroke " OR "cerebrovascular Disorders" OR "brain ischemia" OR "brain haemorrhage" OR "cerebrovascular accident" OR "intracerebral haemorrhage" OR "subarachnoid haemorrhage" OR "transient ischemic attack" OR "brain attack" OR "cerebral embolism" OR "cerebral thrombosis" OR "cerebral haemorrhage" OR "cerebrovascular insult" OR "intraparenchymal haemorrhage" OR "intraventricular haemorrhage" OR "cerebral hypoperfusion" OR "brain infarct" OR "cerebral infarct"). 

Data from the articles and the supplementary materials were extracted. Reference lists from the eligible articles were retrieved for further relevant studies.

### Study selection and data extraction

EndNote X9 (Thompson Reuters, Philadelphia, PA, USA) was used to import all titles and abstracts of the identified articles, and duplicated records were removed. Potentially eligible articles were identified through screening of the titles and abstracts. The full texts of the resulting studies were then thoroughly reviewed by two authors (HAM and MI) and the eligibility of each study was decided. Any disagreements between the investigators were solved by consulting with another investigator (MF).

### Data extraction

Collected information included study characteristics (author, study site, study design), number of patients with ischemic stroke or haemorrhagic stroke in COVID-19 cases and their mortality cases, the chronological order of patient admission to hospital based on symptoms (COVID-19 first, ischemic or haemorrhagic stroke first) and the COVID-19 characteristics (number of patients, severity, and mortality).

### Role of the funding source

This study received no external funding.

### Outcomes

The primary outcomes were: (a) global incidence of ischemic and haemorrhagic stroke in COVID-19 patients; (b) mortality rate of ischemic and haemorrhagic stroke in COVID-19; (c) the frequency of symptoms related to hospital admission among COVID-19 patients with ischemic or haemorrhagic stroke; (d) risk factors of ischemic and haemorrhagic stroke in COVID-19; (e) association of ischemic and haemorrhagic stroke with COVID-19 severity; and (f) association of ischemic and haemorrhagic stroke with mortality of COVID-19. The possible pathogenesis mechanisms of ischemic and haemorrhagic stroke in SARS-CoV-2 infection were also explained in this review.

### Data synthesis

The global prevalence of ischemic stroke was calculated as the number of COVID-19 patients who experienced ischemia divided by the total number of COVID-19 patients with or without ischemic or hemorrhagic stroke, expressed as frequency (%) and 95% CI. The frequency of symptoms that led to hospital admission was calculated as the total number of COVID-19 patients presenting with either respiratory or neurological symptoms first, divided by the total number of ischemic stroke cases among COVID-19 patients, and expressed as percentage and 95% CI. The mortality rate was calculated as the number of deaths of COVID-19 patients who experienced ischemic stroke divided by the total number of COVID-19 patients who experienced ischemic stroke. The same calculations were performed for hemorrhagic stroke.

### Risk of bias assessment

The Newcastle-Ottawa scale (NOS) ^30^ was used to critically assess the quality of the studies included in the meta-analysis. The Q test was used to evaluate the heterogeneity and potential publication bias of the data gathered from the studies.

### Statistical analysis

The association between ischemic and hemorrhagic stroke and the occurrence of COVID-19 was calculated and expressed as the cumulative odds ratio and 95% CI using the Z test; differences with p < 0.05 were considered to be statistically significant. Heterogeneity among studies was assessed using the Q test, and heterogeneous data were analyzed using a random effects model. Publication bias was assessed using Egger’s test and funnel plots (p < 0.05 was considered to indicate potential for publication bias). The data were analyzed using Review Manager version 5.3 (The Cochrane Collaboration) ^31^.

## Results

### Study eligibility

**Figure 1.**
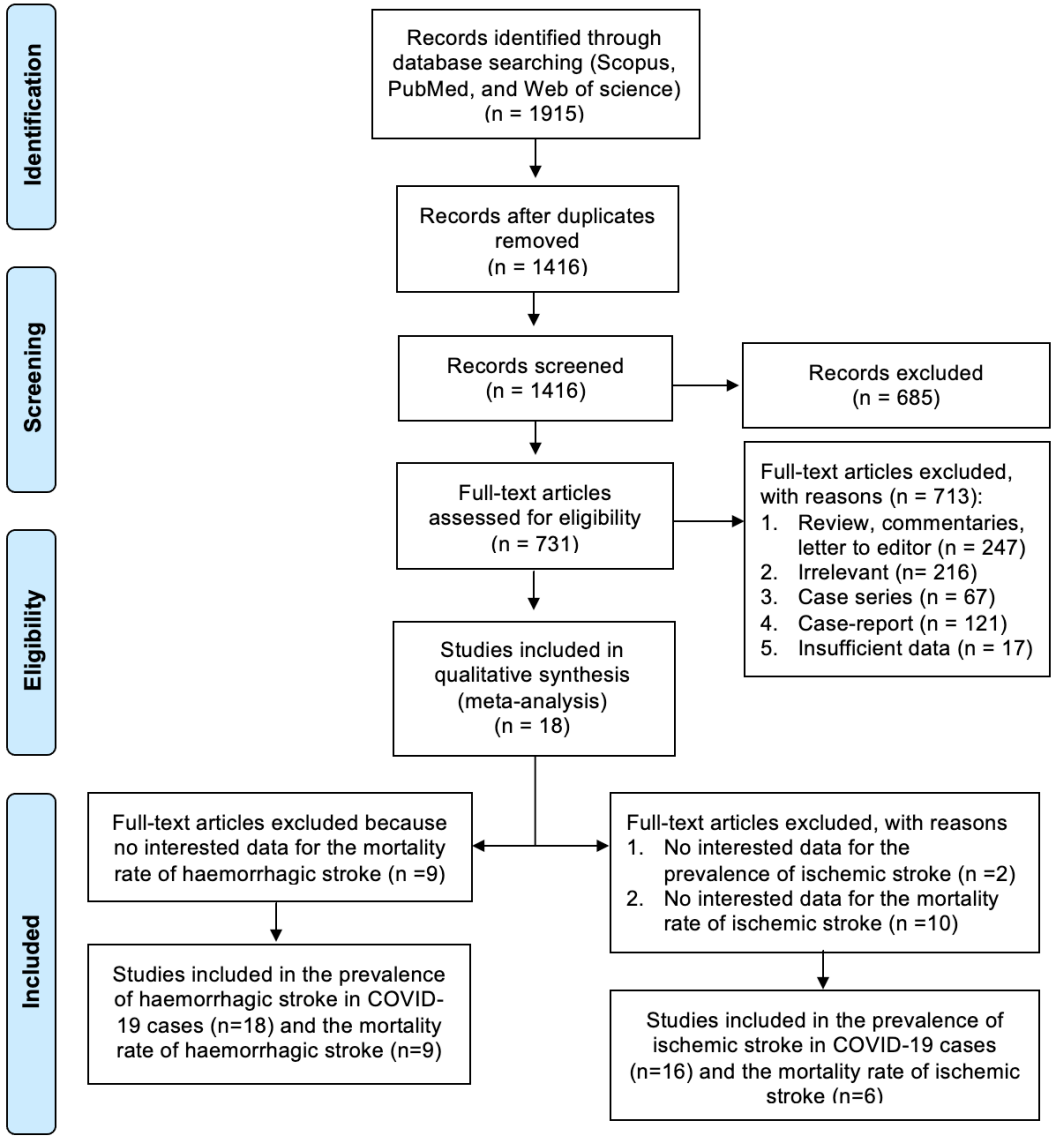
Flowchart of the literature search according to PRISMA.

A total of 1915 articles were retrieved via a literature search, with 1416 citations remaining after the duplicates were removed. An additional 685 articles were excluded after screening the titles and abstracts, leaving 731 studies ( Figure 1), the full texts of which were reviewed for eligibility, with an additional 713 excluded. Exclusions included reviews, irrelevant studies, case series, case reports, and studies with insufficient data. This process resulted in 18 studies being included in the final analysis.

All 18 studies were included in the meta-analysis to calculate the global prevalence of hemorrhagic stroke in COVID-19 patients and the frequency of symptoms leading to hospital admission ^32– 49^. Data from nine studies were included to calculate the mortality rate of hemorrhagic stroke in COVID-19 patients ^32, 33, 35– 37, 40, 45, 48, 49^, while the other nine studies did not report relevant data. The 18 studies and the prevalence of hemorrhagic stroke reported in each of them are summarized in Table 1.

**Table 1.** The prevalence of haemorrhagic stroke among COVID-19 patients around the globe.

Study Design	Country	NOS	COVID-19			Chronology of the symptoms			Ref
			Haemorrhagic Stroke		Total COVID-19	Not clear (%)	Respiratory symptoms first (%)	Neurologic symptoms first (%)	
			Case	Death					
Retrospective cohort	US	8	35	16	5227	35 (16.2)	-	-	32
Retrospective cohort	US	8	33	14	3824	-	29 (13.4)	4 (1.85)	33
Prospective cross- sectional	Spain	7	1	-	2000	-	1 (0.46)	-	34
Retrospective cohort	Spain	8	5	2	1683	-	4 (1.85)	1 (0.46)	35
Retrospective cohort	US	8	9	5	3218	-	5 (2.31)	4 (1.85)	36
Retrospective cohort	UEA	8	12	4	591	12 (5.55)	-	-	37
Retrospective cohort	Turkey	7	2	-	239	2 (0.93)	-	-	38
Retrospective cohort	US	8	14	-	10,596	14 (6.48)	-	-	39
Retrospective cohort	China	8	1	1	219	-	1 (0.46)	-	40
Retrospective cohort	US	8	1	-	509	1 (0.46)	-	-	41
Retrospective cohort	Italy	7	2	-	213	2 (0.93)	-	-	42
Retrospective cohort	US	8	8	-	650	-	2 (0.93)	6 (2.78)	43
Retrospective cohort	Italy	8	11	-	1760	11 (5.09)	-	-	44
Retrospective cohort	UK	8	14	4	3403	14 (6.48)	-	-	45
Retrospective cohort	Multi- national	8	27	-	17,799	27 (12.5)	-	-	46
Retrospective cohort	US	7	3	-	90	-	3 (1.39)	-	47
Retrospective cohort	Multi- national	8	28	16	14,483	28 (12.96)	-	-	48
Retrospective cohort	China	7	10	4	651	10 (4.63)	-	-	49
Total			216	66	67,155	156/216 (72.2)	45/216 (20.83)	15/216 (6.94)	

NOS = Newcastle-Ottawa scale score, COVID-19 = coronavirus disease 19

Only 16 studies were included in the meta-analysis to calculate the global prevalence of ischemic stroke in COVID-19 patients and the frequency of symptoms leading to hospital admission ^34– 49^. Data from six studies were included to calculate the mortality rate of ischemic stroke in COVID-19 patients ^35– 37, 40, 48, 49^, as no relevant data was reported in the remaining 12 studies. The prevalences of ischemic stroke reported in each of these studies are summarized in Table 2.

**Table 2.** The prevalence of ischemic stroke among COVID-19 patients around the globe.

Study design	Country	NOS	COVID-19			Chronology of the symptoms			Ref.
			Ischemic stroke		Total COVID-19	Not clear (%)	Respiratory symptoms (%)	Neurology symptoms (%)	
			Case	Death					
Prospective cross- sectional	Spain	7	10	-	2000	-	6 (1.10)	4 (0.74)	34
Retrospective cohort	Spain	8	17	5	1683	17 (3.13)	-	-	35
Retrospective cohort	US	8	26	10	3218	-	9 (1.65)	17 (3.13)	36
Retrospective cohort	UEA	8	19	4	591	19 (3.49)	-	-	37
Retrospective cohort	Turkey	7	7	-	239	7 (1.29)	-	-	38
Retrospective cohort	US	8	72	-	10,596	72 (13.2)	-	-	39
Retrospective cohort	China	8	10	5	219	-	7 (1.29)	3 (0.55)	40
Retrospective cohort	US	8	7	-	509	7 (1.29)	-	-	41
Retrospective cohort	Italy	7	2	-	213	2 (0.37)	-	-	42
Retrospective cohort	US	8	12	-	650	-	7 (1.29)	5 (0.92)	43
Retrospective cohort	Italy	8	37	-	1760	37 (6.80)	-	-	44
Retrospective cohort	UK	8	6	-	3403	6 (1.10)	-	-	45
Retrospective cohort	Multi- national	8	123	-	17,799	123 (22.6)	-	-	46
Retrospective cohort	US	7	1	-	90	-	1 (0.18)	-	47
Retrospective cohort	Multi- national	8	156	54	14,483	156 (28.68)	-	-	48
Retrospective cohort	China	7	39	18	651	39 (7.17)	-	-	49
Total			544	96	58,104	485/544 (89.1)	30/544 (5.5)	29/544 (5.3)	

NOS = Newcastle-Ottawa scale score, COVID-19 = coronavirus disease 19

### Prevalence of hemorrhagic and ischemic stroke in COVID-19 patients

**Figure 2.**
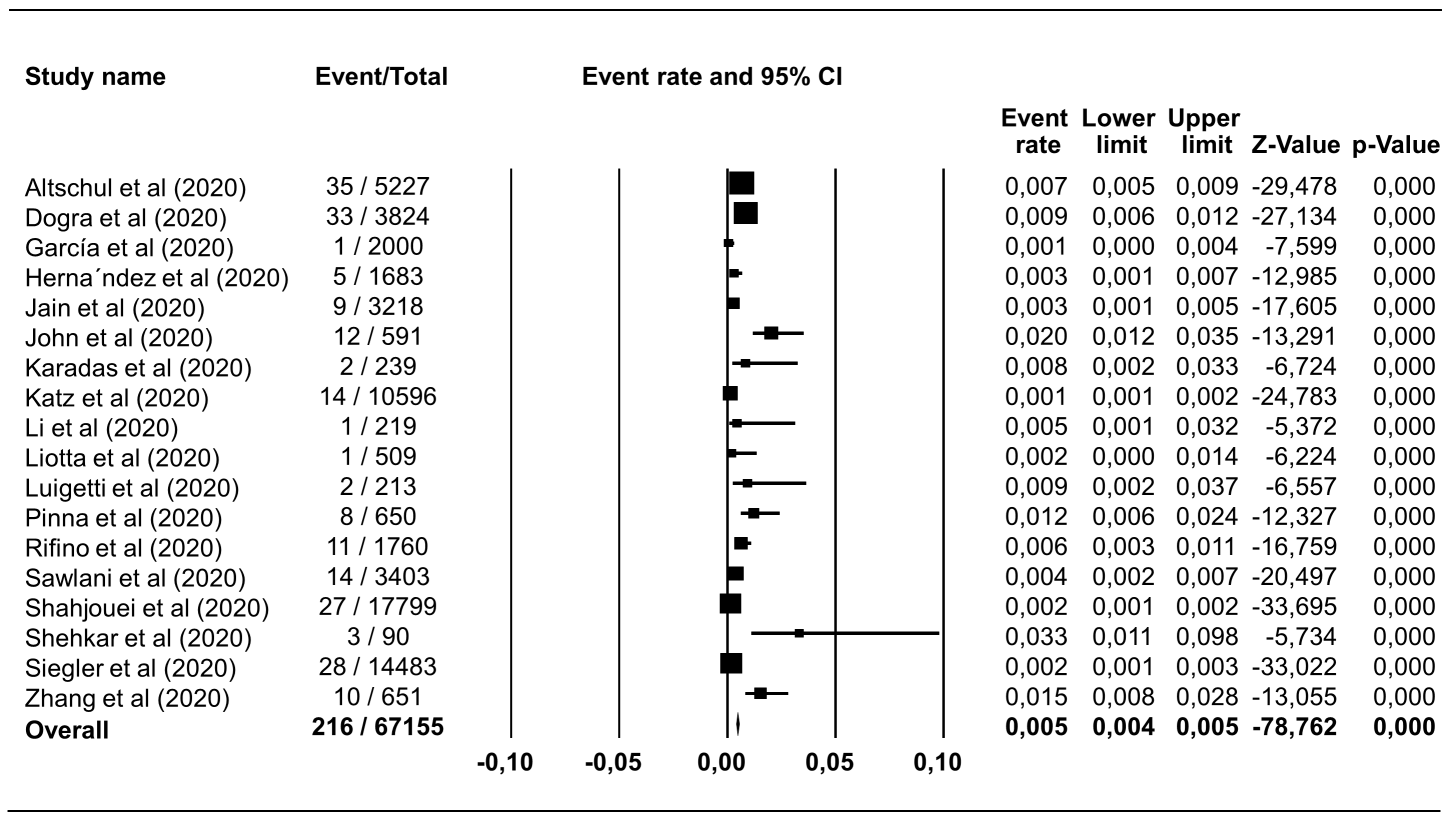
Forest plot of the prevalence of haemorrhagic stroke in COVID-19 patients. The pooled estimate of haemorrhagic stroke prevalence is 0.46% with 95% CI 0.40%–0.53%, p<0.0001; p-value for Egger and heterogeneity is 0.882 and <0.0001, respectively with *I ^2^* 89.81%.

**Figure 3.**
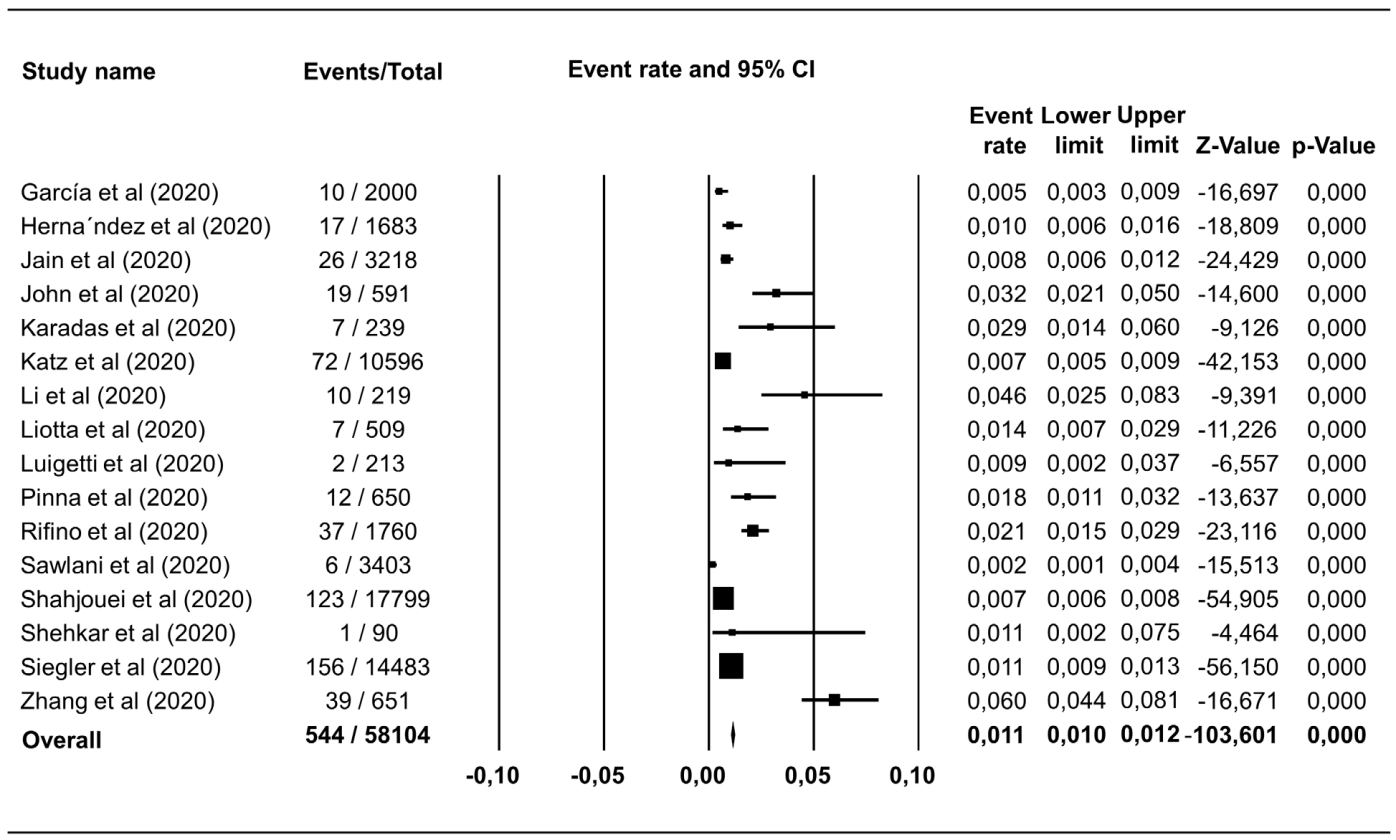
Forest plot of the prevalence of ischemic stroke in COVID-19 patients. The pooled estimate of ischemic stroke prevalence is 1.11% with 95% CI 1.03%–1.22%, p<0.0001; p-value for Egger and heterogeneity is 0.730 and <0.0001, respectively with *I ^2^* 94.07%.

Hemorrhagic stroke was reported in 18 studies including 67,155 COVID-19 patients, with a pooled estimate of prevalence of 0.46% (95% CI 0.40%–0.53%), *I ^2^*=89.81% ( Figure 2). Ischemic stroke was identified in 544 of 58,104 COVID-19 patients in 16 studies, which corresponded to a pooled prevalence estimate of 1.11% (95% CI 1.03%–1.22%), *I ^2^*=94.07% ( Figure 3). The incidence of hemorrhagic and ischemic stroke in COVID-19 patients was 28.42% (216/760) and 71.58% (544/760), respectively.

Among COVID-19 patients who experienced hemorrhagic stroke, 45 of 216 patients (20.83%; 95% CI 15.41%–26.24%) complained of respiratory symptoms before neurological symptoms, while neurological symptoms preceded respiratory symptoms in 15 of 216 patients (6.94%; 95% CI 3.55%–10.33%). No clear onset of either neurological or respiratory symptoms in COVID-19 patients was reported in 11 studies (156/216, 72.22%) ( Table 1).

Among COVID-19 patients who experienced ischemic stroke, 30 of 544 patients (5.51%; 95% CI 3.59%–7.43%) experienced respiratory symptoms before neurological symptoms, while 29 of 544 patients (5.33%; 95% CI 3.44%–7.21%) complained of neurological symptoms before respiratory symptoms. No clear onset of either neurological or respiratory symptoms in COVID-19 patients was reported in 11 studies (485/544, 89.15%) ( Table 2).

### Mortality rate of hemorrhagic and ischemic stroke in COVID-19 patients

**Figure 4.**
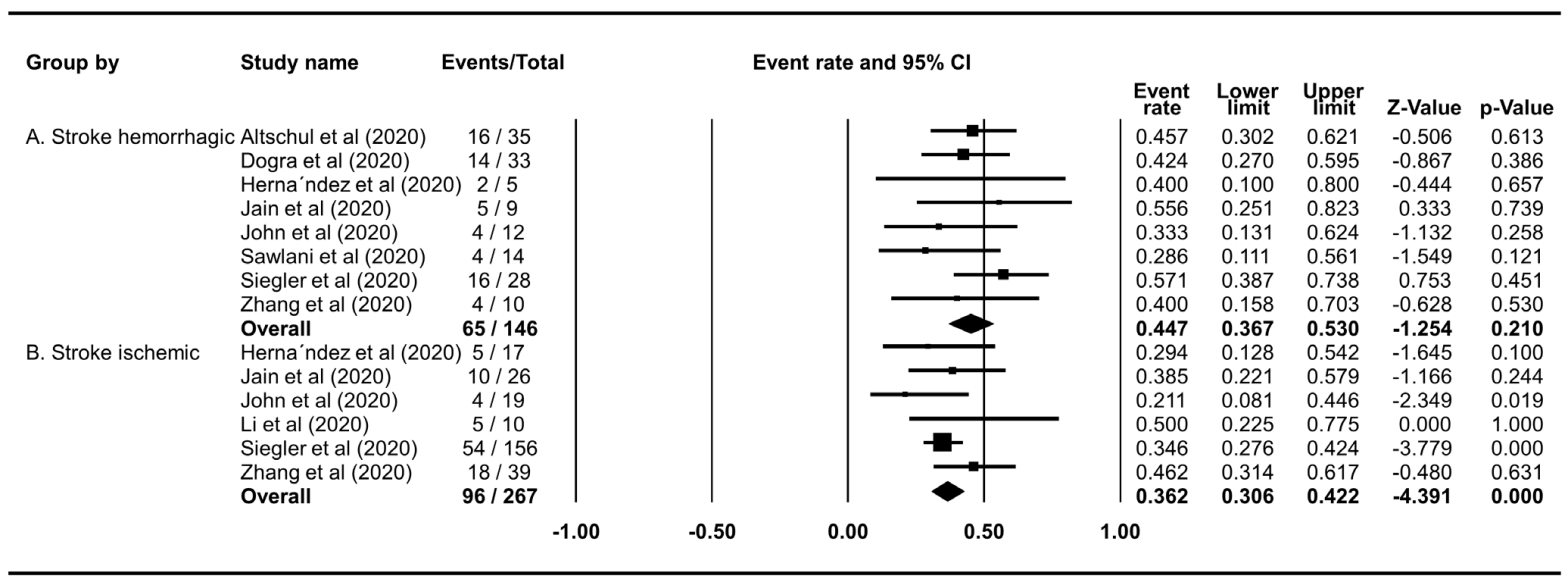
Forest plot of the mortality rate of haemorrhagic and ischemic stroke in COVID-19 patients. ( **A**) The pooled mortality rate of haemorrhagic stroke is 44.72% (95% CI 36.73%–52.98%, p=0.2099); heterogeneity p=0.7343, *I ^2^*=0%, and Egger’s p=<0.0001. ( **B**) The pooled mortality rate of ischemic stroke is 36.23% (95% CI 30.63%–42.24%, p<0.0001); heterogeneity p=0.438, *I ^2^* 0%, and Egger’s p<0.0001.

The mortality rate of COVID-19 patients who experienced hemorrhagic stroke was 44.72% (95% CI 36.73%–52.98%), and the rate was slightly lower in those who experienced ischemic stroke (36.23%; 95% CI 30.63%–42.24%) ( Figure 4).

### Association between hemorrhagic and ischemic stroke and the severity and mortality of COVID-19

There was a lack of data regarding the association between hemorrhagic and ischemic stroke and the severity of the disease and mortality rate of patients with COVID-19. One study reported that 63% of COVID-19 patients who experienced ischemic stroke required admission to the intensive care unit (ICU) ^50^.

## Discussion

### Hemorrhagic stroke in COVID-19 patients

The cumulative incidence of hemorrhagic stroke in COVID-19 patients in the present study was 0.3% (216 of 67,155). This result is lower than a previously reported incidence (0.7%; 95% CI 0.50%–0.9%) ^51^. The incidence of hemorrhagic stroke among all stroke types in COVID-19 patients fluctuated, with 12.2% in May 2020 ^52^, 9.7% in June 2020 ^53^, 17.2% in July 2020 ^54^, 11.6% in September 2020 ^55^, and 28.42% in the present systematic review.

Among 216 COVID-19 patients who experienced hemorrhagic stroke, 20.83% were admitted to a hospital owing to respiratory symptoms and developed a brain hemorrhage during hospitalization, while 6.94% (15/216) were admitted owing to neurological symptoms. The disease course is important for predicting the severity of the systemic disease as COVID-19 in the former group was more severe with abnormal vital sign(s), elevated inflammatory and coagulopathy markers, altered mental status, and the patients likely required mechanical ventilation and ICU care ^43^. Diffuse microhemorrhages have been observed previously in COVID-19 patients, via brain imaging, and such microhemorrhages are scattered mostly in the juxtacortical white matter, corpus callosum, and brain stem ^56– 61^. The fatality rate of COVID-19 patients who experienced hemorrhagic stroke was 44.72% in our study, which is slightly lower than the rate reported previously (48.6%) ^51^.

### Ischemic stroke in COVID-19 patients

The pooled prevalence of ischemic stroke among COVID-19 patients in our systematic review was 0.94% (544 of 58,104). This result is much lower than that described in another report in which acute ischemic stroke was observed in 1.6% of the patients who presented with or were hospitalized owing to COVID-19 ^62^. Another study also reported increased incidence of ischemic stroke among COVID-19 patients compared to that in the non-COVID-19 group (81.4% *vs.* 74.6%) ^63^. The incidence of ischemic stroke in our investigation was significantly lower compared with that in another study (71.58% *vs.* 87.4%, respectively) ^55^.

COVID-19 patients who were admitted with respiratory symptoms and later experienced acute brain infarct comprised 5.51% (30 of 544) of the sample. Meanwhile, 5.33% were admitted owing to neurological symptoms related to ischemic stroke and had a positive result on real-time polymerase chain reaction (RT-PCR) testing for SARS-CoV-2 after screening; there was no clear explanation for hospital admission in the remainder (89.15%). This result is lower than that reported in a previous study, in which ischemic stroke was the reason for hospital admission in 26% of the COVID-19 patients ^62^.

The cause of ischemic stroke is multifactorial in those with SARS-CoV-2 infection, and it may be due to systemic embolization and diffuse microvascular thrombosis (attributed to a significant increase in prothrombotic factors) ^53^. In our systematic review, the mortality rate of COVID-19 patients who experienced ischemic stroke was 36.23%. This rate is higher than that in two previous studies, which reported inpatient mortality rates of 32% and 22.8% ^50^. among COVID-19 patients who experienced ischemic stroke.

### Possible pathogenesis of hemorrhagic stroke in COVID-19 patients

Hemorrhagic stroke is caused by the rupture of cerebral vessels, leading to the extravasation of blood components into the surrounding brain tissue. However, the molecular mechanism by which SARS-CoV-2 infection causes hemorrhagic stroke remains unclear. The ACE2 receptor occupied by the virus appears to be the primary culprit, which then induces subsequent damage to host cells ^64^. Dysfunction of the ACE2 receptor is linked to the elevation of Ang II levels. Ang II is produced from Ang I, and this reaction is catalyzed by the action of ACE. Ang II-related effects are generated after being bound to the AT1 receptor. To counteract the dangerous effects caused by the excessive level of ACE/Ang II/AT1R axis, the ACE2/Ang (1-7)/Mas axis is activated ^65^.

***SARS-CoV-2 infection increases Ang II levels.*** SARS-CoV-2 uses the ACE2 receptor as a portal to enter host cells ^66^. Along with a protease, i.e., TMPRSS2, this receptor assists the virus in infecting cells ^66^. Viral occupation of the ACE2 receptor affects the normal physiological function of the receptor, which is to degrade Ang II, resulting in the accumulation of Ang II in the blood. Elevated Ang II levels are associated with damage linked to the occurrence of hemorrhagic stroke ^67^.

A previous study proposed four modes of action used by Ang II to exert its effects (i.e., direct impact on the vascular system) causing vasoconstriction, promotion of platelet aggregation, increased free radical production, and a reduction in insulin sensitivity ^68^. These actions of Ang II are associated with the occurrence of hemorrhagic stroke. Vasoconstriction is a vital physiological alteration that occurs in hypertension, which is recognized as one of the major risk factors for hemorrhagic stroke ^67^. Ang II is also related to the activation of thrombogenic factors. This may explain the elevation of D-dimer levels, which are monitored in COVID-19 patients, particularly in those with severe infection ^69, 70^. Consequently, the activation of a procoagulant state may induce a hemorrhagic stroke ^71^. Ang II is also known to inhibit the PI3K/AKT signaling pathway, which regulates the secretion of insulin, leading to lowered insulin sensitivity ^68, 72^, which is another risk factor for hemorrhagic stroke. A study using a rat model confirmed that diabetes could degrade tight junction (TJ) proteins mediated by the action of matrix metalloproteinases (MMPs) ^73^. The correlation between junctional disruption, MMPs, and hemorrhagic stroke is described in the next section.

***SARS-CoV-2 infection causes a cytokine storm that induces degradation of the extracellular matrix.*** When SARS-CoV-2 infects the body, the immune system produces massive amounts of pro-inflammatory cytokines in response. An excessive amount of pro-inflammatory cytokines, including tumor necrosis factor-alpha (TNF-α), interleukin (IL)-1β, and IL-6, has been reported in most COVID-19 patients ^74^. This phenomenon—known as the “cytokine storm”—results in the failure of multiple organs and contributes to COVID-19-related death. Ang II is strongly linked to the activation of nicotinamide adenine dinucleotide phosphate (NADPH) oxidase, which is responsible for the development of oxidative stress, a condition that has been known to be correlated with the excessive production of pro-inflammatory cytokines ^67, 75^. An *in vitro* study using a human BBB model revealed that the spike protein of SARS-CoV-2 could elevate the levels of IL-1β and IL-6 ^76^. Several mechanisms have been proposed to explain the role of these cytokines in weakening vessel walls and the subsequent increase in the risk of hemorrhagic stroke, including its effect in the degradation of the extracellular matrix (ECM), which is the primary structure responsible for maintaining the integrity of vascular endothelial cells.

Degradation of the ECM caused by MMPs increases BBB permeability, promotes extravasation of blood components, and contributes to hemorrhagic brain injury ^77^. Many studies have reported that TNF-α can induce the production of MMPs, which are proteolytic enzymes that degrade the ECM. For example, a study reported that TNF-α administered intravenously to mice produced an elevation in the MMP-9 levels, followed by a significant increase in BBB permeability ^78^. Another study demonstrated that MMP-3 expression was upregulated in porcine choroid plexus epithelial cells, which was followed by a reduction in transepithelial electrical resistance, indicating decreased cellular tightness ^79^. Another pro-inflammatory cytokine, IL-1β, is also involved in the induction of MMPs, resulting in the destruction of the ECM. The expression and activity of MMP-2 in cardiac microvascular endothelial cells were induced by IL-1β ^80^. After experimenting with chondrocytes, a study has also revealed that IL-1β exposure leads to MMP-1 upregulation ^81^. This action is suggested to involve various signaling pathways (i.e., ERK1/2, JNKs) and protein kinase C (PKC) ^80– 82^. Studies have also reported the upregulation of MMP expression and activity in various models after exposure to IL-6. This cytokine increases MMP-9 activity during aortic aneurysms and ruptures in mice ^83^. MMP-2 and MMP-9 levels have also been found to be increased in COVID-19 patients, which is consistent with the elevation of IL-6 expression in patients with lymphoma ^84^. A STAT3 signaling pathway has been proposed as the pathway used by IL-6 to upregulate MMPs ^83, 85^.

Moreover, the impairment of ECM caused by those pro-inflammatory cytokines may be significantly associated with the action of reactive oxygen species (ROS), such as superoxide and singlet oxygen, and reactive nitrogen species, such as nitrogen oxide and peroxynitrite ^86, 87^. A study found that TNF-α and IL-6 administration in human brain microvascular endothelial cells (HBMVEC) induced increased levels of ROS ^75^. Oxidative stress-related BBB disruption leading to the incidence of stroke is strongly related to the activation of MMPs ^88– 91^.

Interestingly, MMPs could alter the regulation of junctional proteins. A study using a rat model demonstrated that TJ damage in cerebral vessels was mediated by MMP-2 and MMP-9, and that this action could be inhibited by the MMP inhibitor BB-1101 ^92^. A study found that the degradation of occludin, a transmembrane protein of the TJ, in BBB model bEnd3 monolayer was mediated by MMP-2 ^93^, while another study confirmed that MMP-9 mediated the destruction of TJ protein in a BBB model hCMEC/D3 ^94^.

***SARS-CoV-2 infection induces a cytokine storm that causes disturbance in junctional protein formation.*** Elevation of cytokine levels caused by SARS-CoV-2 infection could weaken vessel walls and ultimately increase the incidence of hemorrhagic stroke by impairing cellular junctional proteins, which is also the primary structure responsible for maintaining vascular endothelial cell integrity. The integrity of vascular endothelial cells is, in large part, determined by the presence of junctional proteins. In general, three major junctions are located in the BBB: TJs, adherens junctions (AJs), and gap junctions ^95^. Any disturbances occurring in any of these structures will ultimately lead to vascular endothelial dysfunction. A previous study proposed that serum levels of TJ proteins may be used to predict the incidence of a hemorrhagic event(s) following ischemic stroke ^96^.

Disruption of junctional proteins could be caused by pro-inflammatory cytokines. Using bEnd.3 endothelial cells as the BBB model, a previous study demonstrated that TNF-α and IL-6 exposure produced a significant increase in cellular permeability, which could be attributed to the decreased expression of ZO-1 and claudins ^97^. These findings are supported by a study investigating primary cerebral microvessels isolated from sheep, which revealed that 100 ng/mL of IL-6 reduced the expression of occludin ^98^. The expression of cadherin, occludin, and claudin-5 led to a dose-dependent decrease in a HBMVEC model after treatment with TNF-α and IL-6 ^75^. Using human umbilical vein endothelial cells (HUVECs), another study reported that the expression of occludin and E-cadherin was downregulated following exposure to interferon (IFN)-γ ^99^. TNF-α treatment to HUVECs caused a change in localization of claudin-5 and JAM-A, while this cytokine also reduced the expression of occludin ^100^.

Damage to junctional proteins could also be associated with the disruption of polarity complex proteins. Polarity proteins work by regulating many aspects of cellular differentiation and proliferation, including junctional protein formation and localization ^101, 102^. Although the understanding of polarity complexes is mainly supported by a wide range of experiments involving epithelial cells, the complexes also play pivotal roles in endothelial cells ^103, 104^. Thus, impairments to polarity complexes could subsequently result in damage to transmembrane and cytoplasmic junctional proteins. For example, PATJ knockdown Caco2 cells affect the localization of occludin and ZO-3 in TJ formation ^105^. Another study reported that VE-cadherin, the major transmembrane protein of AJ, connected to Pals1 during the formation of vascular lumen indicating the specific role of this polarity protein in regulating junctional formation in endothelial cells ^106^. Interestingly, SARS-CoV-2 has been suggested to interact with the Pals1 protein in host cells through its envelope (E) protein ^107^. A study demonstrated that another betacoronavirus—SARS—also uses this mode of interaction with host cells ^108^.

The action of cytokines in downregulating junctional proteins could be mediated by the activation of NADPH oxidase ^75^. This enzyme is one of the main sources of ROS in the vascular system, along with mitochondrial enzymes and xanthin oxidase ^109^. It should be noted that the activation of NADPH oxidase is also linked to endothelial dysfunction leading to COVID-19-related thrombotic events ^110^. It has been proposed that SARS-CoV-2 induces a thrombosis event by stimulating various tissue factors that are dependent on the activation of NADPH oxidase following its attack on endothelial cells ^111^.

Collectively, hemorrhagic stroke in COVID-19 patients may be associated with the elevation of Ang II levels, which is an event subsequent to SARS-CoV-2 occupation of the ACE2 receptor. The cytokine storm is also responsible for the degradation of some important components of cerebral vessels, such as MMPs and TJ, triggering cerebral vascular rupture.

### Possible pathogeneses of ischemic stroke in COVID-19 patients

SARS-CoV-2 infection could cause ischemic stroke through the induction of a hypercoagulative state, endothelial injury, cytokine storm, and/or cardiogenic embolism ^21^. Dysfunction of endothelial cells (induced by SARS-CoV-2 infection) may increase thrombin formation and fibrinolysis ^112^. Coagulopathy due to a thrombosis event has been observed in COVID-19 patients, with elevated D-dimer and fibrinogen, although with no significant prolonged prothrombin time and activated partial thromboplastin time ^25, 26, 113^. Increased fibrinogen levels also contribute to hyperviscosity, which is consistently found in COVID-19 patients, in whom viscosity varies between 1.9 to 4.2 centipoise (normal range, 1.4–1.8 centipoise) ^23^. Hyperviscosity is not only caused by increases in fibrinogen level, but also by the cytokine storm, which plays an important role in increasing viscosity levels in those with COVID-19 by inducing the excessive release of IL-6 and TNF-α ^114, 115^.

Systemic inflammation, which activates the complement pathway, induces the excessive release of inflammatory cytokines, causing venous thromboembolism by platelets and also inducing a hypercoagulative state ^116– 118^. This hypercoagulability could lead to macro- and microthrombus formation, which ultimately leads to cerebrovascular incidents ^119, 120^.

The ACE2 receptor also plays an important role in the neurological manifestations of SARS-CoV-2 infection. ACE2 converts Ang II into ANG (1-7), which plays an essential role as a neuroprotector. Administration of ANG (1-7) in animal models resulted in a decrease in neurological deficits and infarct size in rats with ischemic stroke ^121, 122^. Therefore, in COVID-19, the SARS-CoV-2 spike protein binds to the ACE2 receptor, resulting in decreased ANG (1-7) synthesis ^123^. Cardioembolic (19.21%) and atherothrombotic (7.39%) events have also been reported to contribute to the etiology of ischemic stroke in COVID-19 patients ^124^.

## Conclusion

Although the prevalence of hemorrhagic and ischemic stroke is low in COVID-19 patients, this systematic review may increase awareness among clinicians regarding the potentially high mortality rate of individuals with this infection who experience a stroke, especially those with severe infection.

## Data availability

### Underlying data

All data underlying the results are available as part of the article and no additional source data are required.

### Reporting guidelines

Figshare: PRISMA checklist for ‘Hemorrhagic and ischemic stroke in patients with coronavirus disease 2019: Incidence, risk factors, and pathogenesis - A systematic review and meta-analysis’, https://doi.org/10.6084/m9.figshare.13513509 ^28^.

Data are available under the terms of the Creative Commons Attribution 4.0 International license (CC-BY 4.0).
