# Supplementary material for: Hemorrhagic and ischemic stroke in patients with coronavirus disease 2019: incidence, risk factors, and pathogenesis - a systematic review and meta-analysis
Source: F1000Res. 2021 Jan 19;10:34. [Version 1] doi: 10.12688/f1000research.42308.1 (PMC7934095; doi:10.12688/f1000research.42308.1)
Supplement: Peer review history

# Peer review: Reviewer response for version 1

**DOI:** 10.5256/f1000research.45374.r80516

**Published:** 2021-05-24

**Authors:** Mahir Gachabayov

**Affiliations:** 1 Department of Surgery, Westchester Medical Center, Valhalla, NY, USA

This is a well-conducted systematic review and a well-written manuscript evaluating the prevalence, clinical presentations, and mortality rates of hemorrhagic and ischemic stroke among COVID-19 patients. In addition, the authors have attempted to describe the pathogenesis behind stroke in such patients.

 The research question makes sense. The objective of this systematic review was to shed light on a question with limited evidence in the current literature. The Introduction is well written and details the gap and controversy in the current literature.

 The protocol of the review was prospectively registered in PROSPERO. No deviations from the original protocol were observed. The report complies with the PRISMA guidelines. The search strategy is comprehensive and was reported in detail. Major databases were utilized as data sources. The methodology of eligibility screening and study selection, quality assessment, and data extraction is adequate. Statistical methods are adequate as well.

 Results are well-presented in tables summarizing included studies and forest plots. The prevalence of hemorrhagic and ischemic strokes were found to be low at 0.5% and 1%, respectively. Nonetheless, the mortality rates of these entities were quite high at 45% and 36%. These findings are clinically relevant and add to the literature.

 Discussion is comprehensible. Interpretation of the statistical findings is logical and the conclusions are justified.

Are the rationale for, and objectives of, the Systematic Review clearly stated?

Yes

Is the statistical analysis and its interpretation appropriate?

Yes

Are sufficient details of the methods and analysis provided to allow replication by others?

Yes

Are the conclusions drawn adequately supported by the results presented in the review?

Yes

Reviewer Expertise:

Clinical outcomes and Evidence Synthesis

I confirm that I have read this submission and believe that I have an appropriate level of expertise to confirm that it is of an acceptable scientific standard.

# Peer review: Reviewer response for version 1

**DOI:** 10.5256/f1000research.45374.r80518

**Published:** 2021-03-04

**Authors:** Guilherme Welter Wendt

**Affiliations:** 1 Faculty of Medicine, Western Paraná State University, Francisco Beltrão, Brazil

This is the report of a registered systematic review (SR; https://www.crd.york.ac.uk/prospero/display_record.php?RecordID=224470). The authors correctly deployed the methodology proposed, in addition to adding a few more objectives - such as the chronological order of symptoms. In summary, the study not only updated a previous SR on this specific topic but largely expanded the mechanisms underpinning both ischemic and hemorrhagic stroke. The authors also followed the PRISMA guidelines and assessed risk of bias using a well-known scale. The statistical analyses are sound and based on best practices for conducting SR and meta-analyses.

 Some questions and suggestions that emerged while reading the paper include:

- It would be sensible to adopt American (i.e., hemorrhagic) or British (i.e., haemorrhagic) English in the manuscript, including subheadings and figures.
- The flowchart presents “excluded studies” in the last stage, in which authors are supposed to present studies that were included. I suggest a revision of this last step to avoid confusion.
- It is not clear if all patients were diagnosed by RT-PCR as suggested in the registration (CRD42020224470). I assume this was done at some stage during the screening/study eligibility stages. Nonetheless, if suspected cases were also included, this information needs to be clearly stated.
- Was there any cut-off score or other criteria for including/excluding studies due to risk of bias assessments?
- Figures 2 to 4 are misspelling “Hernández”.
- In the discussion, I think it is always a good idea to be precise as possible. As such, I would always refer to COVID-19 patients when making comparisons (i.e., “This result is lower than a previously reported incidence”, which appears in the first paragraph of the discussion).
- In the second paragraph of the discussion, when the authors mention 216 COVID-19 patients, are we really relying on RT-PCR confirmed cases? In other passages, the authors are more clear about the method of diagnosis (“Meanwhile, 5.33% were admitted owing to neurological symptoms related to ischemic stroke and had a positive result on real-time polymerase chain reaction (RT-PCR) testing for SARS-CoV-2 after screening; there was no clear explanation for hospital admission in the remainder (89.15%)”.
- The authors could briefly expand or give examples of neurological symptoms. For instance, the next sentence says altered mental status, albeit I do believe more objective symptoms would be of extreme value.

Other than that, I believe the contribution is original, relevant and has substantial contribution to the field.

Are the rationale for, and objectives of, the Systematic Review clearly stated?

Yes

Is the statistical analysis and its interpretation appropriate?

Yes

Are sufficient details of the methods and analysis provided to allow replication by others?

Yes

Are the conclusions drawn adequately supported by the results presented in the review?

Yes

Reviewer Expertise:

Quantitative research methods; Systematic reviews and meta-analyses; Epidemiology; Public health.

I confirm that I have read this submission and believe that I have an appropriate level of expertise to confirm that it is of an acceptable scientific standard.

# Peer review: Reviewer response for version 1

**DOI:** 10.5256/f1000research.45374.r77660

**Published:** 2021-02-24

**Authors:** Ismail Setyopranoto

**Affiliations:** 1 Department of Neurology, Universitas Gadjah Mada, Yogyakarta, Indonesia

This article is very good and relevant to the conditions of the COVID-19 pandemic, maybe it only needs to add differences in imaging examinations, namely head CT scans between intracerebral hemorrhages due to degenerative diseases, for example due to hypertension and those caused by COVID-19 infection.

 I agree that this article can be indexed with some additions that I have written above.

Are the rationale for, and objectives of, the Systematic Review clearly stated?

Yes

Is the statistical analysis and its interpretation appropriate?

Yes

Are sufficient details of the methods and analysis provided to allow replication by others?

Yes

Are the conclusions drawn adequately supported by the results presented in the review?

Yes

Reviewer Expertise:

Neurologist

I confirm that I have read this submission and believe that I have an appropriate level of expertise to confirm that it is of an acceptable scientific standard.
